
JOURNAL INFORMATION
==============================
NLM Title Abbreviation: ScientificWorldJournal
Journal ID: ScientificWorldJournal
NLM Journal ID: 101131163
Journal ID: TSWJ

The Scientific World Journal

ISSN: 2356-6140
EISSN: 1537-744X
Publisher: Wiley

ARTICLE INFORMATION
==============================
PMCID: PMC5763746
PMID: 20209383
DOI: 10.1100/tsw.2010.51
Article ID: 245868
Article version: 1
Subjects: Clinical Images

Granuloma Annulare

Gupta Diptesh 1 *
Hess Brian 2
Bachegowda Lohith 3
1 Department of Internal Medicine, University of Missouri – Columbia, MO, USA
2 MU School of Medicine, Columbia, MO, USA
3 Department of Internal Medicine, Drexel University, Philadelphia, USA
*Diptesh Gupta: guptadi@health.missouri.eduAcademic Editor: Daniel Batlle

Publication date: 2010
Electronic publication date: 2010 Mar 5
Volume: 10
First page: 384
Last page: 386
Received 2009 Nov 10; Revised 2010 Jan 18; Accepted 2010 Mar 2
Copyright: Copyright © 2010 Diptesh Gupta and Brian Hess.
Copyright year: 2010
License: This is an open access article distributed under the Creative Commons Attribution License, which permits unrestricted use, distribution, and reproduction in any medium, provided the original work is properly cited.
License URL: https://creativecommons.org/licenses/by/3.0/

Keywords: erythematous rash, granuloma annulare, diabetes mellitus

==============================
Clinical Image
*TheScientificWorldJOURNAL* (2010) 10, 384–386
ISSN 1537-744X; DOI 10.1100/tsw.2010.51

TheScientificWorldJOURNAL
www.thescientificworld.com

### Granuloma Annulare

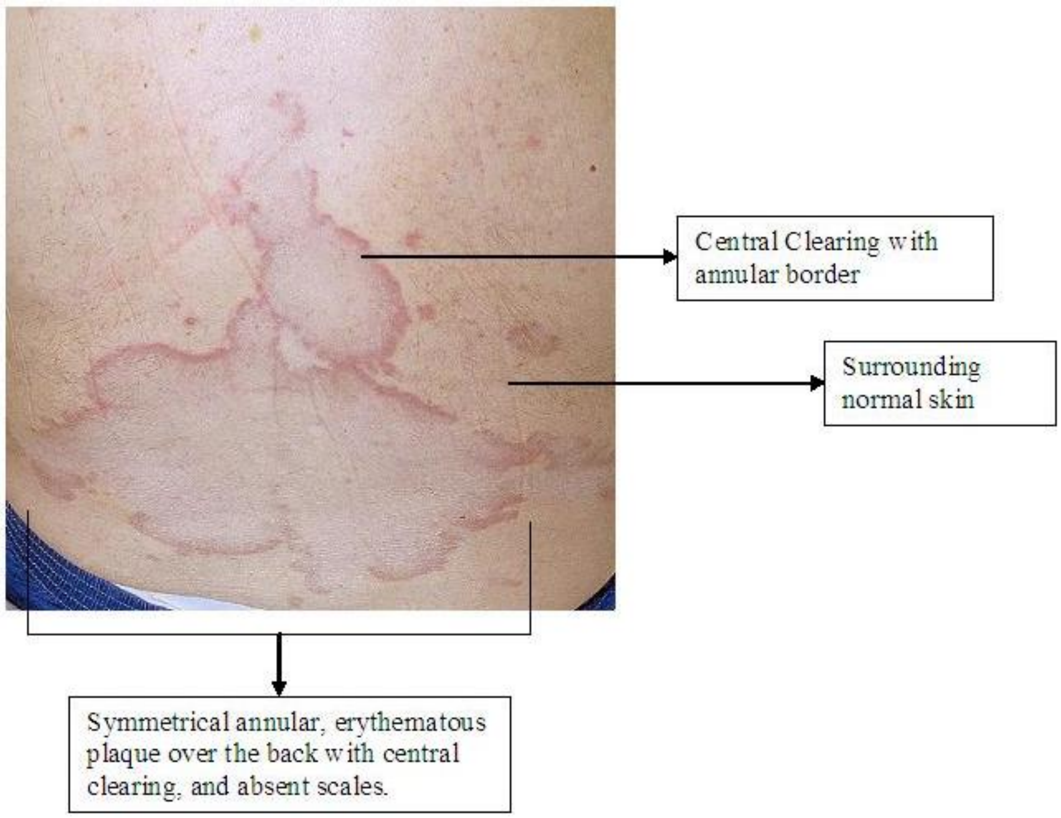

**FIGURE 1.** Back of the patient.

Diptesh Gupta[1,]*, Brian Hess[2], and Lohith Bachegowda[3]
*[1]Department of Internal Medicine, University of Missouri – Columbia; [2]MU School of Medicine, Columbia, MO; [3]Department of Internal Medicine, Drexel University, Philadelphia*

**E mail**: guptadi@health.missouri.edu; hessb@missouri.edu; Lohith.Shakaldevanapura@DrexelMed.edu

*Received November 10, 2009; Revised January 18, 2010; Accepted March 2, 2010; Published March 5, 2010*

**KEYWORDS**: erythematous rash, granuloma annulare, diabetes mellitus

*Corresponding author.
©2010 with author.
Published by TheScientificWorld; www.thescientificworld.com

We present a case of a 77-year-old, diabetic male with a 20-year history of a migratory erythematous, asymptomatic, generalized, nonscaly, and nonitchy rash that started over the dorsum of his left hand. On examination, there were multiple annular erythematous plaques, distributed symmetrically and diffusely over his torso and arms, with central clearing and no scales (Figs. 1–3). He was empirically treated with topical antifungals with no improvement. A potassium hydroxide (KOH) microscopic slide was prepared, which yielded a negative result for a fungal infection. A punch biopsy of the skin helped us to arrive at the diagnosis of a generalized granuloma annulare (GA).

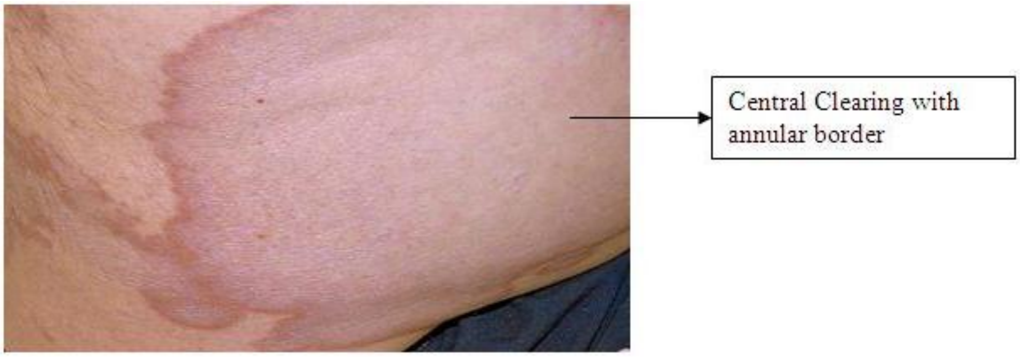

**FIGURE 2.** Right flank.

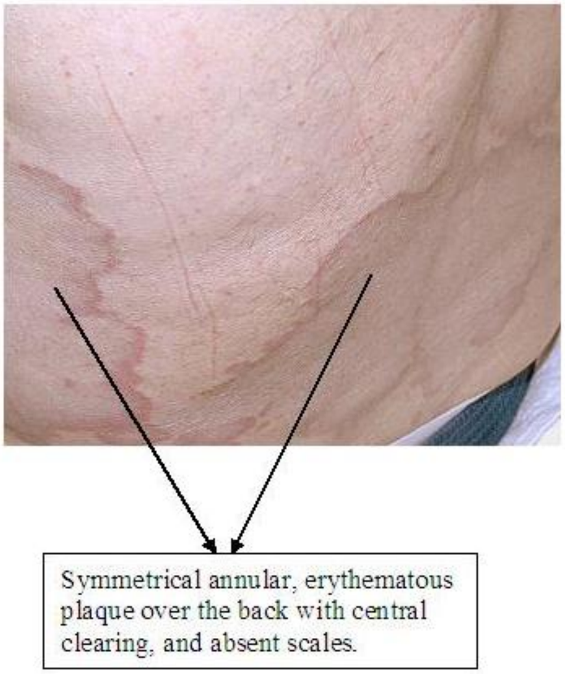

**FIGURE 3.** Left flank. Note the symmetrical distribution of the rash.

GA is a benign, self-limiting skin condition of unknown etiology that is often asymptomatic, but can rarely be associated with mild pruritus[1]. Women are twice as likely to have GA as compared to men[2]. The cause of this condition is unknown, but it has been associated with diabetes mellitus, infections such as HIV, and malignancies such as lymphoma. These lesions typically start as a ring of flesh-colored papules that slowly progress with central clearing[1]. Lack of symptoms, scaling, or associated vesicles helps to differentiate GA from other skin conditions such as tinea corporis, pityriasis rosea, psoriasis, or erythema annulare centrifugum[4,5]. The KOH slide preparation and a punch biopsy helped to confirm the diagnosis[3,4]. Treatment is often not needed as the majority of these lesions are self-resolving within 2 years[1]. Treatment may be pursued for cosmetic reasons. Available options include high-dose steroid creams, PUVA, cryotherapy, or drugs such as niacinamide, infliximab, Dapsone, and topical calcineurin inhibitors[1,5].

### REFERENCES

1. Cyr, P.R. (2006) Diagnosis and management of granuloma annulare. *Am. Fam. Physician* **74(10),** 1729–1734.
2. Friedman, S.J. and Winkelmann, R.K. (1987) Familial granuloma annulare. Report of two cases and review of the literature. *J. Am. Acad. Dermatol.* **16(3 Pt 1),** 600–605.
3. Smith, M.D., Downie, J.B., and DiCostanzo, D. (1997) Granuloma annulare. *Int. J. Dermatol.* **36(5),** 326–333.
4. Barron, D.F., Cootauco, M.H., and Cohen, B.A. (1997) Granuloma annulare. A clinical review. *Lippincotts Prim. Care Pract.* **1(1),** 33–39.
5. Setterfield, J., Huilgol, S.C., and Black, M.M. (1999) Generalised granuloma annulare successfully treated with PUVA. *Clin. Exp. Dermatol*. **24(6),** 458–460.

**This article should be cited as follows:**

Gupta, D., Hess, B., and Bachegowda, L. (2010) Granuloma annulare. *TheScientificWorldJOURNAL* **10**, 384–386. DOI 10.1100/tsw.2010.51.
